# Mitigating SSIs: focus on physical operation rooms environmental factors

**DOI:** 10.1017/ash.2024.241

**Published:** 2024-09-16

**Authors:** Lakshmi Medepalli, Mohamed Yassin, Heather Dixon, Mathea Schafer

**Affiliations:** Pitt Public Health; University of Pittsburgh; UPMC

## Abstract

**Background:** Surgical site infections (SSIs) are associated with increased morbidity, monetary loss and mortality. The physical aspects of the operation room (OR) including airflow, humidity, pressure, and particulate counts are essential part of SSI prevention. Humidity control is vital to avoid static electricity buildup.Temperature control helps prevent hypothermia. Limiting OR traffic and door opening are essential to prevent airflow disturbance and minimze particles in OR environment. We have recently studied electronic monitoring of OR traffic and the traffic was higher than what was expected. Our aim was to evaluate our real-life measurement of these OR parameters as part of SSI prevention bundle. **Methods:** This is a prospective study focused on the OR physical environmental factors as part of operative SSI prevention bundle. The study was conducted for 4 weeks at an academic medical center. The study was conducted in two different generations of OR for neurosurgical and ophthalmologic procedures. We performed direct observation of OR traffic as well as environmental parameters (temperature, humidity, pressure, and particulate count) for the entire length of the procedure. We used both directly measured data as well as automated data generated by facilities. **Results:** The study showed that temperature, humidity, and pressure wer tightly controlled in the OR. This observation was consistent between manual data and automatically generated data. The OR traffic was not easily monitored by the current automatic data and was measured by direct observation. The correlations between particulate count and OR traffic was strongest for 0.3μm (0.7370, and weakest for 1.0μm ( 0.087). The 5.0μm particulate size had a moderate positive correlation of 0.344, Additionally, shorter procedures had less particulate matter in the OR environment. Automated data were only available in the new ORs but could not predict traffic without automated door monitors. But the automated data could easily portray the temperature, humidity and pressure minute by minute. **Conclusion:** OR traffic increases the particle count particularly the small size. Other physical aspects of the OR environment were tightly controlled. The ability to automatically monitor OR parameters could be extremely helpful for assuring patient safety as well as reviewing OR factors in SSI cases.

